# Clinical impact of deep-vein thrombosis screening in recipients of lung transplants: A contemporary single-center study

**DOI:** 10.1016/j.xjon.2026.101979

**Published:** 2026-07-30

**Authors:** Jose Ramirez, Shrikar Modukuri, Kenyon Agrons, Marcelo Salan-Gomez, Andres Leon-Pena, Jason Thomas, Colton Andrews, Allen Kuncheria, Cyrus Vahdatpour, Senthil Sukumar, Muhammad Hayat Syed, Subhasis Chatterjee, Gabriel Loor, Anh Nguyen, Nirmal Sharma, Erik E. Suarez, Ramiro Fernandez

**Affiliations:** a Division of Cardiothoracic Transplantation and Circulatory Support, Baylor College of Medicine, Houston, Tex; b Division of Pulmonary Medicine, Department of Medicine, Baylor College of Medicine, Houston, Tex; c Section of Hematology/Oncology, Department of Medicine, Baylor College of Medicine, Houston, Tex

**Keywords:** lung transplantation, venous thromboembolism, pulmonary embolism

## Abstract

**Objective:**

Venous thromboembolism (VTE) events are common after lung transplantation and can have a significant impact on morbidity and mortality. Our objective was to assess the impact of a deep-vein thrombosis (DVT) screening program on VTE incidence and clinical outcomes in recipients of lung transplants.

**Methods:**

We conducted a single-center retrospective study of all adults who received lung transplants (≥18 years) at our center between May 2020 and June 2024, excluding multiorgan transplants. Patients were divided into prescreening (May 2020 to June 2022) and screening (June 2022 to June 2024) groups. We evaluated VTE incidence, graft function, bleeding complications, and survival.

**Results:**

There were a total of 287 patients in the study, 130 in the prescreening group and 157 in the screening group. DVT incidence was significantly greater in the screening group compared with the prescreening group (54.1% vs 20.7%; *P* < .001). There was no difference in the incidence of pulmonary embolus, graft function by percent expiratory volume in 1 second predicted out to 1 year, overall survival out to 1 year, nor bleeding complications between groups.

**Conclusions:**

DVT screening in recipients of lung transplants detects clinically insignificant DVTs and may introduce potential harm from overtreatment. The use of DVT-screening programs in recipients of lung transplants should be reconsidered.

**Figure:**
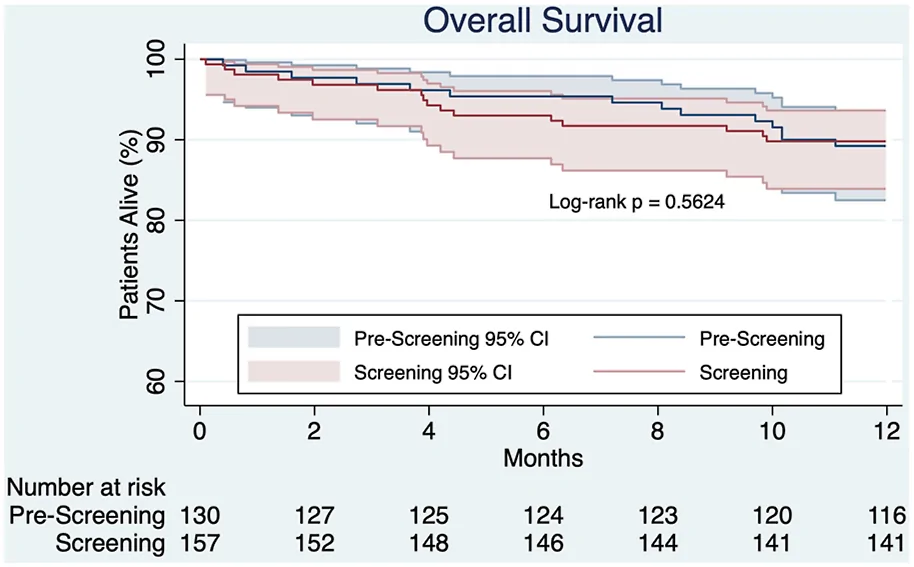
There was no difference in overall survival between the screening and prescreening groups.

**Central Message**

Routine DVT screening in recipients of lung transplants detects clinically insignificant DVTs.

**Perspective**

Recipients of lung transplants are at a high risk of VTE events. Here, we evaluated the outcomes of a DVT-screening program in a high-volume transplant center, which doubled the incidence of DVT without improving the incidence of pulmonary embolism, graft function, nor survival. DVT screening in recipients of lung transplants detects clinically insignificant DVT with potential for overtreatment.

Lung transplantation remains the standard of care for patients with end-stage lung disease.^1^ However, despite decades of progress in surgical technique and perioperative care, recipients of transplanted lungs continue to experience the worst survival of all recipients of solid organs.^1^ Accordingly, there is a persistent drive to enhance care of recipients of transplanted lungs and improve posttransplant outcomes.

Despite contemporary prophylaxis strategies, recipients of lung transplant continue to experience a high incidence of venous thromboembolism (VTE) events, affecting up to 64% of patients during the index hospitalization after transplant.^2^, ^3^, ^4^, ^5^, ^6^, ^7^ Because bronchial circulation is not restored during transplantation, early allograft viability depends primarily on pulmonary arterial blood flow, rendering recipients of lung transplants vulnerable to the consequences of VTE including graft necrosis, failure, and increased mortality.^3^^,^^8^

Given the high incidence of VTE events in recipients of lung transplants, with a limited ability to reduce this risk, several transplant centers have initiated deep-vein thrombosis (DVT)-screening programs for the early detection and treatment of DVTs.^2^^,^^7^^,^^8^ However, the clinical impact of DVT screening in recipients of lung transplants remains uncertain. Only 1 previous study, by Jorge and colleagues,^2^ evaluated DVT-screening outcomes, with the authors reporting a nonsignificant trend toward improved 1-year survival. However, it remains unknown whether DVT screening impacts the incidence of pulmonary embolism (PE), graft function, or survival. Furthermore, it is unknown whether DVT screening leads to overtreatment and potential harm from therapeutic anticoagulation. The potential risks and benefits of DVT screening should be fully evaluated before universal implementation is recommended. In this study, we evaluated the impact of a DVT-screening program in recipients of lung transplants on the incidence of PE, graft function, survival, and anticoagulation morbidity at a high-volume lung transplant program.

## Methods

### Study Population

We maintain an observational registry with prospectively collected data on all lung transplants performed at Baylor St Luke's Medical Center, approved by Baylor College of Medicine's institutional review board (no. H-41620; approved April 8, 2021) with a waiver of consent. We included all adult recipients of lung transplants (age ≥18 years) at Baylor St Luke's Medical Center between May 2020 and June 2024, to encompass 2 years before and after implementation of the DVT-screening program. Patients who received multiorgan transplants were excluded. Patients were divided into prescreening and screening groups.

### DVT-Screening Program and Prophylaxis Measures

A DVT-screening program was implemented in June 2022 after 2 sentinel PE events that lead to donor lung necrosis and listing for redo transplant. The goal of the screening program was for early detection and management of DVT to prevent VTE-related morbidity and mortality. Upper- and lower-extremity duplex ultrasonography was performed on postoperative day (POD) 1 and again the week before discharge. Of note, the POD1 screening primarily detects pre-existing DVT. By screening before discharge, we capture the entire index hospitalization period. Before the screening program, DVT testing was based on clinical suspicion. In both prescreening and screening eras, workup for PE was based on clinical suspicion. Routine computed tomography scans to screen for PE were not performed.

Therapeutic anticoagulation was the preferred treatment for diagnosed DVT regardless if detection was by screening or by clinical suspicion. However, clinical conditions may have resulted in an alternate treatment strategy, such as inferior vena cava filter or observation. For patients who were treated with therapeutic anticoagulation, a heparin or bivalirudin infusion was initiated and subsequently converted to apixaban at discharge. Therapeutic anticoagulation continued for 3 to 6 months for patients diagnosed with a DVT during the index stay, with confirmation of DVT resolution on follow-up duplex ultrasound before the discontinuation of therapy. Patients who developed a PE were managed with therapeutic anticoagulation indefinitely. The screening group also underwent administration of 5000 units of subcutaneous heparin before induction at the time of transplant. Postoperatively, all patients underwent standard DVT prophylaxis measures, including sequential compression device use starting on POD 0 and chemoprophylaxis starting on POD2 with either 5000 units of subcutaneous heparin twice daily or 40 mg subcutaneous enoxaparin once daily. With the implementation of the screening program, Lovenox (enoxaparin) became the preferred chemoprophylaxis strategy. Antiplatelet agents were not routinely administered posttransplant.

### Outcomes of Interest

We evaluated the effect of the screening program on VTE incidence, graft function by percent forced expiratory volume in 1 second (FEV1%) predicted, recipient survival, and bleeding events. Recipients of lung transplants remain at risk of VTE beyond the index hospitalization. Therefore, PE incidence was also evaluated through 1-year posttransplant to assess the downstream effects of increased therapeutic anticoagulation use.

### Statistical Analyses

Statistical analyses were conducted using StataBE 18.0 (StataCorp LLC). Continuous variables were summarized as median with interquartile range and compared using the Mann-Whitney *U* test. Shapiro-Wilk test was used to assess normality for continuous variables. Categorical variables were summarized as counts with percentages and compared using the χ^2^ or Fisher exact test, as appropriate.

Multivariable models were constructed to evaluate factors associated with outcomes of interest, using logistic regression for binary outcomes and Cox proportional hazards modeling for time-to-event outcomes. Candidate covariates were selected a priori on the basis of clinical relevance and previous literature, including demographic characteristics, comorbidities, and operative variables, rather than through automated or stepwise selection methods, to minimize overfitting and enhance interpretability. All selected variables were entered simultaneously into the models. Collinearity was assessed using variance inflation factors before inclusion, and model parsimony was considered in relation to the number of outcome events.

For logistic regression models, discrimination was evaluated using the area under the receiver operating characteristic curve (c-statistic), and calibration was assessed using the Hosmer-Lemeshow goodness-of-fit test. For Cox models, discrimination was evaluated using Harrell's C-statistic, and the proportional hazards assumption was assessed using Schoenfeld residuals. All statistical tests were 2-sided.

## Results

### Lung Recipient Characteristics

A total of 287 lung transplant recipients were included, with 130 in the prescreening group and 157 in the screening group (Table 1). Baseline characteristics were similar between groups. The screening group included a greater proportion of female recipients (54.8% vs 40.0%; *P* = .01), whereas age, body mass index, and primary lung diagnosis did not differ. As a marker of acuity, rates of pretransplant hospitalization and venovenous extracorporeal membrane oxygenation (ECMO) bridging were similar; however, intraoperative extracorporeal life support (ECLS) use was more frequent in the screening group (61.8% vs 46.9%; *P* = .016).

**Table 1:** Characteristics of recipients of lung transplants

Characteristics	Prescreening (n = 130)	Screening (n = 157)	*P* value
Age, y	61.0 (50.0-68.0)	61.0 (53.0-68.0)	.40
Sex			.01
Female	52 (40.0%)	86 (54.8%)	
Male	78 (60.0%)	71 (45.2%)	
BMI	26.5 (22.3-31.6)	25.7 (22.5-29.5)	.23
Primary diagnosis			.21
Cystic fibrosis	5 (3.8%)	2 (1.3%)	
Obstructive lung disease	26 (20.0%)	37 (23.6%)	
Pulmonary vascular disease	3 (2.3%)	9 (5.7%)	
Restrictive lung disease	96 (73.8%)	109 (69.4%)	
Redo lung transplant	8 (6.2%)	17 (10.8%)	.235
Condition at time of transplant			1.00
Hospitalized, not in ICU	7 (5.4%)	11 (7.0%)	
ICU	21 (16.2%)	30 (19.1%)	
ECMO bridge to transplant	17 (13.1%)	17 (10.8%)	.69
Intraoperative ECLS use	61 (46.9%)	97 (61.8%)	.016
Type of lung transplant			.064
Bilateral	95 (73.1%)	130 (82.8%)	
Single	35 (26.9%)	27 (17.2%)	
Chemoprophylaxis strategy			.004
Heparin SQ	78 (60.0%)	64 (40.8%)	
Enoxaparin SQ	52 (40.0%)	93 (59.2%)	

Data presented as median (interquartile range) or n (%). *BMI*, Body mass index; *ICU*, intensive care unit; *ECMO*, extracorporeal membrane oxygenation; *ECLS*, extracorporeal life support; *SQ*, subcutaneous.

### Incidence and Distribution of VTE

The overall incidence of DVT during index hospitalization after transplant more than doubled from 20.7% (27/130) in the prescreening group to 54.1% (85/157) in the screening group (*P* < .001) (Table 2). The incidence of DVT did not differ on the basis of chemoprophylaxis strategy (Table E1). The rate of symptomatic DVTs was similar between groups: 20.7% (27/130) versus 19.1% (30/157) in the prescreening and screening groups, respectively. This suggests the increase in DVT incidence was from detection of incidental, ie, asymptomatic, DVT. Despite the greater than 2-fold increased incidence of DVT in the screening group compared with the prescreening group, the incidence of PE did not differ between groups during index hospitalization, 2.3% (3/130) versus 1.3% (2/157), *P* = .66, or at 1-year posttransplant, 9.2% (12/130) versus 6.4% (10/157); *P* = .49.

**Table 2:** Venous thromboembolism incidence and treatment

Venous thromboembolism incidence and treatment	Prescreening (n = 130)	Screening (n = 157)	*P* value
DVT incidence (index hospitalization)	27 (20.7%)	85 (54.1%)	<.001
Symptomatic DVT	27 (20.7%)	30 (19.1%)	.23
PE (index hospitalization)	3 (2.3%)	2 (1.3%)	.66
PE (1-y)	12 (9.2%)	10 (6.4%)	.49
DVT treatment (index hospitalization)			
Anticoagulation	18 (13.8%)	48 (30.6%)	.001
IVC filter	2 (1.5%)	16 (10.2%)	.08
Observation	7 (5.4%)	37 (23.6%)	<.001

Data presented as n (% of patients). *DVT*, Deep-vein thrombosis; *PE*, pulmonary embolism; *IVC*, inferior vena cava.

The distribution of DVTs is shown in Table 3. Overall, there were 46 individual DVTs detected in the prescreening group compared with 135 in the screening group. Between groups, there was a similar DVT distribution across all sites.

**Table 3:** DVT characteristics

DVT characteristics	Prescreening (n = 46)	Screening (n = 135)	*P* value
Upper proximal DVT	22 (47.8%)	67 (49.6%)	.87
Upper distal DVT	3 (6.5%)	11 (8.1%)	1.00
Both upper proximal and distal	8 (17.4%)	11 (8.1%)	.10
Lower proximal DVT	8 (17.4%)	31 (23.0%)	.54
Lower distal DVT	5 (10.9%)	10 (7.4%)	.54
Both lower proximal and distal	0	5 (3.7%)	.33

Data presented are total number of DVTs diagnosed during the study period. Upper proximal DVT includes axillary, subclavian, and internal jugular veins. Upper distal DVT includes the brachial vein. Lower proximal DVT includes femoral and popliteal veins. Lower distal DVT includes soleal, tibial, peroneal, and gastrocneumius veins. *DVT*, Deep-vein thrombosis.

Next, we performed a subanalysis to assess whether intraoperative ECLS was associated with the development of lower-extremity DVT, because our preferred ECLS strategy includes femoral vein cannulation (Table E2). On multivariate logistic regression analysis, intraoperative ECLS was not associated with lower-extremity DVT (odds ratio, 1.81; 95% CI, 0.85-3.84; c-statistic, 0.71). Obesity was the sole risk factor independently associated with lower-extremity DVT (odds ratio, 2.62; 95% CI, 1.31-5.24). Similarly, we performed a subanalysis of factors associated with upper-extremity DVT (Table E3; c-statistic, 0.59). Notably, ECMO bridge to transplant and PICC placement were not associated with upper-extremity DVT.

### DVT Characteristics in Patients With PE

Table E4 describes DVT characteristics in patients who developed PE within 1-year posttransplant. In the prescreening group, there were 12 patients who developed PE, and none received anticoagulation before the PE diagnosis. In the screening group, there were 10 patients with PE and 3 received previous anticoagulation. These 3 patients had screening-detected DVT during the index stay and were discharged on apixaban. In all 3 cases, the PE occurred in the outpatient setting. Therefore, although most PE occurred in patients who were not anticoagulated, 30% of the cases in the screening group occurred in patients discharged on anticoagulation.

In terms of asymptomatic DVT associated with a PE, there were 55 patients in the screening group with asymptomatic DVT. Seven (12.7%) developed PE; however, 6 were in the outpatient setting. Of those 6 patients who developed PE as an outpatient, the asymptomatic DVT had resolved by the time of the PE diagnosis.

### Treatment for DVT and Bleeding Complications

The percentage of patients who received therapeutic anticoagulation was higher in the screening group compared with the prescreening group (30.6% vs 13.8%) (Table 2). Inferior vena cava filter use for DVT management was more frequent in the screening group (19.0% vs 7.4%), as was management by observation alone (43.5% vs 25.9%).

Overall bleeding complication rates were similar between groups (3.9% vs 4.5%; *P* = 1.0) (Table 4). Of note, the 4 cases of intracranial hemorrhage in the screening group were not associated with anticoagulation for screening-detected DVT.

**Table 4:** Bleeding complications and survival outcomes

Bleeding complications and survival outcomes	Prescreening (n = 130)	Screening (n = 157)	*P* value
Overall bleeding complications	5 (3.9%)	7 (4.5%)	1.00
Hemothorax	4 (3.1%)	2 (1.3%)	.42
Retroperitoneal hematoma	1 (0.8%)	1 (0.6%)	1.00
Intracranial hemorrhage	0	4 (2.6%)	.13
90-d survival	126 (96.9%)	152 (96.8%)	1.00
1-y survival	116 (89.2%)	141 (89.8%)	1.00

Data presented as n (% of patients).

### Graft Function

We assessed graft function by FEV1% at 3, 6, and 12 months posttransplant. There were no significant differences in median FEV1% between groups at any time point (Figure 1).

**Figure 1:**
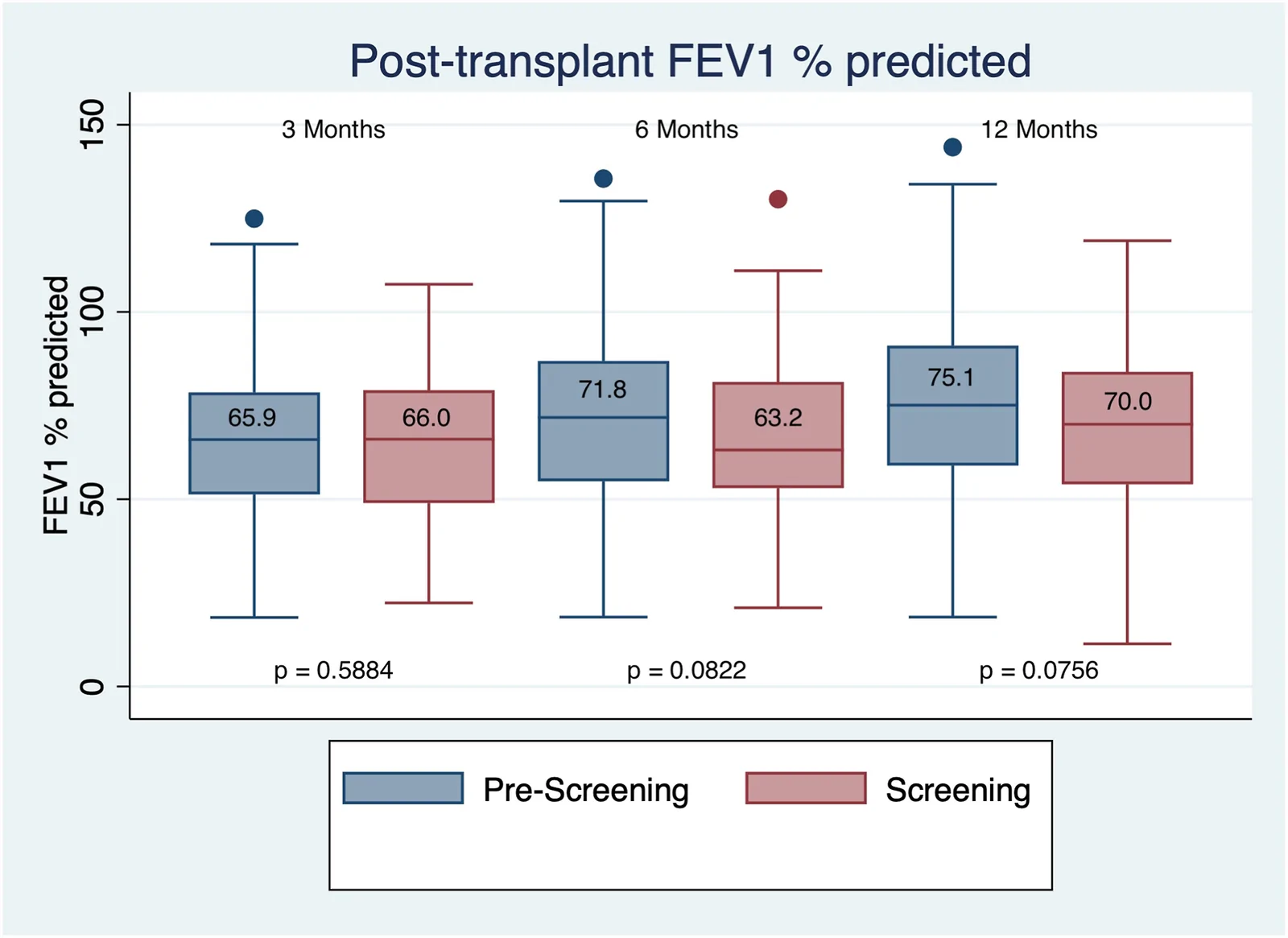
Median percent forced expiratory volume in 1 second (*FEV1%*) predicted at 3, 6, and 12 months posttransplant. The *lower and upper borders of the box* represent the lower and upper quartiles (25th percentile and 75th percentile). The *middle horizontal line* represents the median. The *lower and upper whiskers* represent the minimum and maximum values of nonoutliers. *Extra dots* represent outliers.

### Survival

There was no difference in overall survival between groups (Figure 2). Ninety-day survival was 96.9% in the prescreening group and 96.8% in the screening group (*P* = 1.0), and 1-year survival was 89.2% versus 89.8% (*P* = 1.0). On adjusted Cox proportional hazards modeling, DVT screening was not associated with 90-day (hazard ratio, 0.92; 95% CI, 0.23-3.77, c-statistic: 0.77) nor 1-year mortality (hazard ratio, 1.11; 95% CI, 0.52-2.32, c-statistic: 0.68) (Table 5).

**Figure 2:**
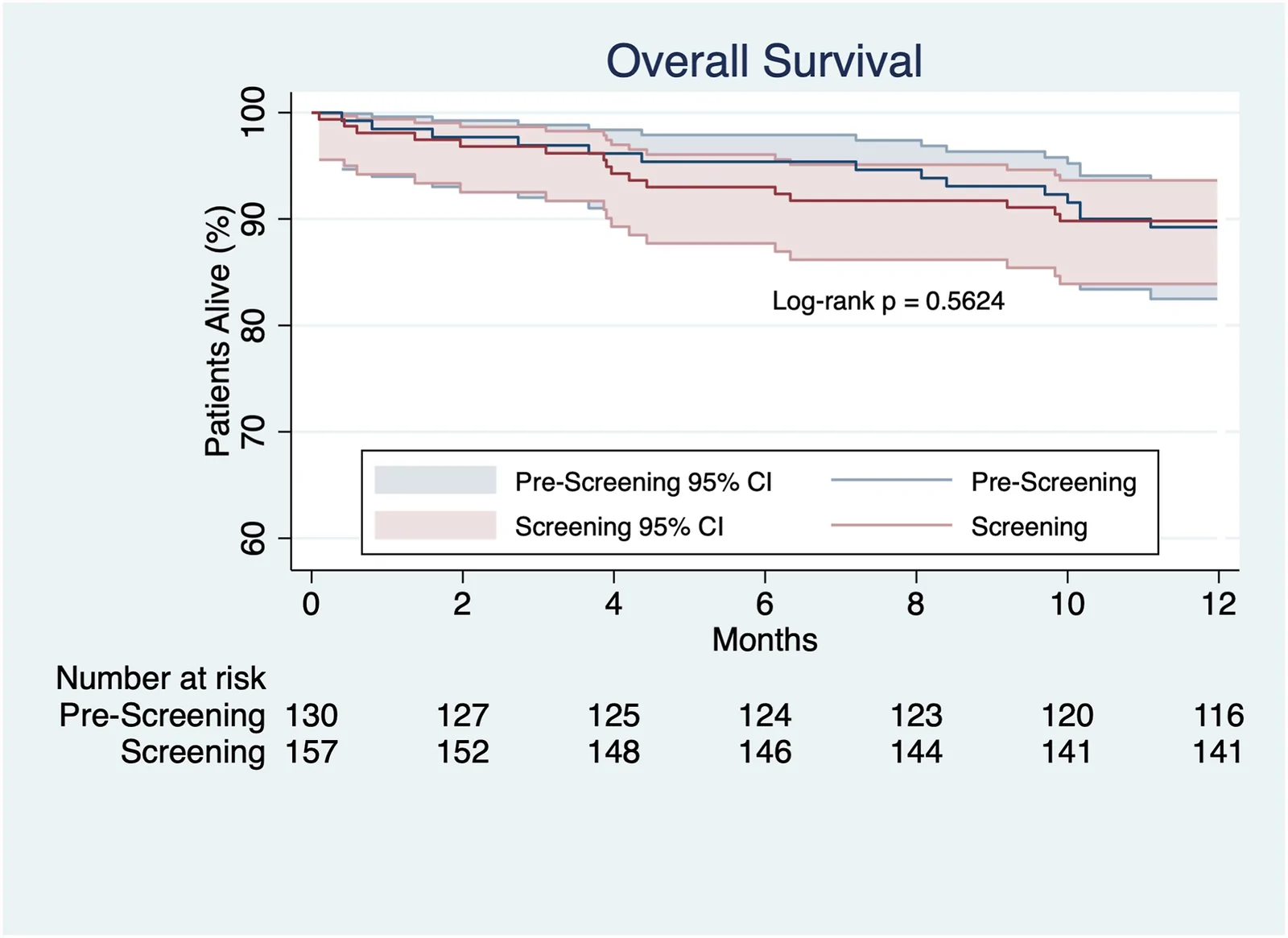
Kaplan-Meier survival curve. 95% CI is depicted.

**Table 5:** Cox proportional hazards model for mortality in screening versus prescreening eras

Variable	Hazards ratio (95% CI)	*P* value
90-d mortality		
DVT screening	0.92 (0.23-3.77)	.91
Male	0.11 (0.01-0.87)	**.037**
Obesity (BMI >30)	0.76 (0.15-3.92)	.74
Restrictive lung disease	0.65 (0.16-2.56)	.54
ECMO BTT	1.56 (0.15-16.00)	.71
Double-lung transplant	1.50 (0.16-13.63)	.72
1-y mortality		
DVT screening	1.11 (0.52-2.35)	.79
Male	0.35 (0.16-0.75)	**.007**
Obesity (BMI >30)	0.89 (0.39-2.06)	.79
Restrictive lung disease	1.65 (0.65-4.17)	.29
ECMO BTT	0.67 (0.15-3.03)	.61
Double-lung transplant	0.57 (0.24-1.36)	.21

*P* values in bold are statistically significant.

*DVT*, Deep-vein thrombosis; *BMI*, body mass index; *ECMO BTT*, extracorporeal membrane oxygenation bridge to transplant.

## Discussion

VTE events are very common in recipients of lung transplants and represent a significant source of morbidity and mortality.^2^^,^^3^^,^^8^ Modern DVT-risk assessments and widespread prophylaxis measures have failed to reduce the burden of VTE events. This has led several lung transplant centers, including our own, to implement DVT-screening protocols for their early detection and treatment in hopes of preventing VTE-related morbidity and mortality. Despite the growing trend, the clinical utility of DVT screening has not been fully assessed in this patient population. In this study, we provide, to our knowledge, the first comprehensive evaluation of a routine DVT screening program at a high-volume lung transplant center. Implementation of screening more than doubled the incidence of DVT detection after transplantation. However, increased detection and treatment of DVT did not improve clinical outcomes. There was no difference in incidence of PE, survival, or graft function. These findings suggest that routine screening identifies clinically insignificant DVTs, leading to potential overtreatment.

VTE is the leading cause of preventable in-hospital mortality, and recipients of lung transplants are at an elevated risk of VTE-related morbidity and mortality.^9^ Because bronchial circulation is not restored in the lung allograft, lung viability is initially completely dependent on pulmonary artery blood flow until collaterals develop. As such, a PE could result in graft loss and a high risk of recipient death. In addition, it is conceivable that DVTs may lead to subclinical PE events that may contribute to lung function decline over time. In this context, it would seem justifiable to implement a DVT-screening program to reduce the risk of potential catastrophic events in recipients of lung transplants. However, screening for asymptomatic DVT has been controversial in other high-risk patient populations. Sinnamon and colleagues^10^ found a potential benefit to postoperative screening for DVT in patients with abdominal malignancies. In their single-center study, they found a reduction in the incidence of PE with early detection and treatment of DVTs. However, evidence supporting DVT screening in nonmalignant cases is mixed. Like the lung transplant population, VTE events are very common in patients with trauma, with a reported incidence between 20% and 58%.^11^^,^^12^ DVT-screening protocols have been studied in the trauma population since the 1990s with mixed results.^11^^,^^13^^,^^14^ A recent meta-analysis found that DVT screening in patients with trauma was associated with a reduced incidence of PE without a clear effect on mortality.^12^ Other studies in both patients with trauma and those undergoing neurosurgery found a significant increase in resource use and costs associated with DVT screening without a significant clinical benefit.^13^^,^^15^ Overall, there remains a lack of consensus on the clinical utility of DVT screening high-risk surgical populations.

The 54.1% incidence of DVT in the screening era in our study is greater than that reported by other groups with DVT-screening programs. The Pittsburgh and Temple groups reported DVT incidence of 17.3% and 24.6%, respectively. However, there are notable differences in the patient populations and screening protocols across our institutions that likely contributed to the differences. The Pittsburgh study had a lower risk recipient population with a lower incidence of restrictive lung disease and intraoperative ECMO use in only 14.3% of cases. In addition, they screened for lower-extremity DVT only.^2^ The Temple group performed single-lung transplants predominantly (68.9% of cases). They screened both upper and lower extremities; however, there was only one screening test performed within 14 days of transplant. Our study included a high-risk recipient population with 70% restrictive lung disease, 20% in the intensive care unit at time of transplant, 10.8% bridged to transplant with ECMO, 61% use of intraoperative ECLS, and bilateral lung transplants in 82.8% of cases. In addition, our screening protocol maximized the chance of DVT detection with both upper- and lower-extremity duplex scans early after transplant and again before discharge.

Inherent to DVT-screening programs is uncertainty regarding the management of asymptomatic DVTs. As the principal VTE management guideline, the most recent CHEST guideline for antithrombotic therapy for VTE disease does not address treatment of asymptomatic DVT.^16^ Because the goal of our DVT-screening program was to prevent VTE-associated morbidity, we preferentially treated asymptomatic screening-detected DVTs with therapeutic anticoagulation unless there were mitigating factors such as elevated bleeding risk. Our study was designed to assess the clinical utility of a DVT-screening program, not specifically to address the question of asymptomatic DVT management. Further studies should examine whether asymptomatic DVTs warrant therapeutic anticoagulation.

Furthermore, a natural question that arises from our study is what DVT factors are most likely to result in PE. Although our study is underpowered to address this question, an analysis of the patients who developed PE in our series may offer some hypotheses. Of the 22 patients who developed PE, most (77.2%) occurred in the outpatient setting. Notably, although all patients had duplex scans done at the time of PE diagnosis, only 8 patients were found to have DVT at that time. Of these, 7 had lower-extremity DVT and 1 had upper- and lower-extremity DVT. Therefore, lower-extremity DVT may be more likely to lead to PE. In addition, although 7 of the 10 patients who developed PE in the screening group had asymptomatic DVT during their index stay, by the time of the PE event the asymptomatic DVT had resolved. This suggests asymptomatic DVT have a low likelihood of progressing to symptomatic PE. More large multicenter studies should determine what DVT features constitute high risk for progression to PE to target treatment to those high-risk DVTs. An additional consideration regarding DVT screening is the impact on hospital quality metrics. VTE is an important quality metric for hospital systems, and increasing the detection of asymptomatic DVTs without improving clinical outcomes may negatively affect hospital quality scores.

This study has several limitations. The conclusions drawn from this study are under the assumption that the true incidence of DVT was similar across both the prescreening and screening eras, with the difference being detection of asymptomatic DVT in the screening era. Because the prescreening era only included symptomatic DVT, the true DVT incidence (including asymptomatic DVT) in that era remains unknown. However, the baseline recipient characteristics and clinical practices at our center remained largely stable during the study period, allowing focused assessment of a single variable–the DVT-screening program. In addition, because we did not perform screening computed tomography scans for PE, the true incidence of PE in this group may be underestimated. The treatment of screening detected DVT was not standardized and therefore subject to selection bias in the treatment decision. Its single-center retrospective design may limit generalizability. Furthermore, the sample size may be insufficient to detect differences in outcomes, in particular the incidence of PE and mortality, which were rare events.

## Conclusions

This study suggests that a DVT-screening program in recipients of lung transplants detects clinically insignificant DVTs and exposes patients to potential overtreatment. Therefore, the use of DVT-screening programs in recipients of lung transplants should be reconsidered.

### Webcast

You can watch a Webcast of this AATS meeting presentation by going to: https://www.aats.org/resources/clinical-impact-of-deep-vein-t-12368.

**Figure:**
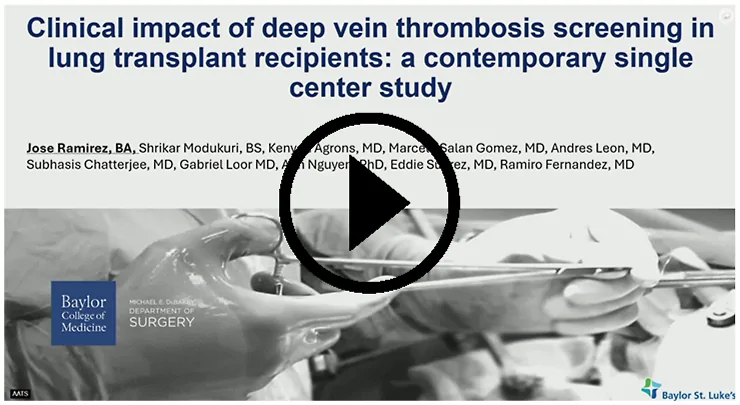

### Audio

You can listen to the discussion audio of this article by going to the supplementary material section below.

## Conflict of Interest Statement

G.L. is consultant for Maquet, cofounder and chief medical officer for Organvive, and former consultant for Transmedics and Abiomed. S.S. is a consultant for Novartis, Alexion, and Sanofi-Genzyme. All other authors reported no conflicts of interest.

The *Journal* policy requires editors and reviewers to disclose conflicts of interest and to decline handling or reviewing manuscripts for which they may have a conflict of interest. The editors and reviewers of this article have no conflicts of interest.

## Figures and Tables

**Table E1:** DVT incidence by chemoprophylaxis strategy

Variable	Heparin SQ	Lovenox SQ	*P* value
DVT incidence	64 (47.1%)	71 (49.0%)	.811

*SQ*, Subcutaneous; *DVT*, deep-vein thrombosis.

**Table E2:** Multivariable logistic regression for lower-extremity DVT

Variable	Odds ratio (95% CI)	*P* value
Intraoperative ECLS	1.81 (0.85-3.84)	.124
Previous lung transplant	1.28 (0.36-4.64)	.703
Condition at the time of transplant		
Not hospitalized	Ref	
Hospitalized, not in ICU	0.47 (0.08-2.77)	.407
ICU	0.40 (0.07-2.15)	.286
Male	1.12 (0.58-2.18)	.736
Obese (BMI >30)	2.62 (1.31-5.24)	.007
Restrictive lung disease	1.98 (0.85-4.62)	.113
ECMO bridge to transplant	2.29 (0.38-13.58)	.360

*DVT*, Deep-vein thrombosis; *ECLS*, extracorporeal life support; *ICU*, intensive care unit; *BMI*, body mass index; *ECMO*, extracorporeal membrane oxygenation.

**Table E3:** Multivariable logistic regression for upper-extremity DVT

Variable	Odds ratio (95% CI)	*P* value
PICC	1.00 (0.60-1.69)	.99
Intraoperative ECLS	0.75 (0.41-1.35)	.33
Previous lung transplant	1.04 (0.40-2.68)	.94
Condition at the time of transplant		
Not hospitalized	Ref	
Hospitalized, not in ICU	0.91 (0.31-2.73)	.87
ICU	0.99 (0.33-2.96)	.99
Male	0.74 (0.44-1.26)	.27
Obese (BMI >30)	0.79 (0.44-1.44)	.45
Restrictive lung disease	1.03 (0.57-1.85)	.93
ECMO bridge to transplant	2.66 (0.75-9.39)	.13

*DVT*, Deep-vein thrombosis; *PICC*, peripherally inserted central catheter; *ECLS*, extracorporeal life support; *ICU*, intensive care unit; *BMI*, body mass index; *ECMO*, extracorporeal membrane oxygenation.

**Table E4:** DVT characteristics of patients with pulmonary embolism within 1 year posttransplant

Variable	Setting	DVT: index stay	DVT location (index)	Symptomatic DVT: index	DVT: outpatient	DVT location (outpatient)	Anticoagulation before PE
Prescreening group							
1	Outpatient	Yes	Proximal upper	Yes	Yes	Proximal lower	No
2	Outpatient	No	N/A	N/A	Yes	Distal lower	No
3	Index	No	NA	NA	N/A	N/A	No
4	Index	No	N/A	N/A	N/A	N/A	No
5	Index	No	N/A	N/A	N/A	N/A	No
6	Outpatient	No	N/A	N/A	No	N/A	No
7	Outpatient	No	N/A	N/A	Yes	Proximal and distal lower	No
8	Outpatient	No	N/A	N/A	No	N/A	No
9	Outpatient	No	N/A	N/A	No	N/A	No
10	Outpatient	No	N/A	N/A	No	N/A	No
11	Outpatient	No	N/A	N/A	Yes	Proximal lower	No
12	Outpatient	No	N/A	N/A	No	N/A	No
Screening group							
13	Index	Yes	Distal lower	No	No	N/A	No
14	Outpatient	Yes	Proximal upper	No	No	N/A	Yes
15	Outpatient	Yes	Distal lower	No	Yes	Distal lower	Yes
16	Index	Yes	Proximal upper and proximal lower	Yes	N/A	N/A	No
17	Outpatient	Yes	Proximal upper	No	No	N/A	No
18	Outpatient	Yes	Proximal upper	No	No	N/A	Yes
19	Outpatient	Yes	Proximal upper	No	No	N/A	No
20	Outpatient	No	N/A	N/A	No	N/A	No
21	Outpatient	No	N/A	N/A	Yes	Proximal and distal lower	No
22	Outpatient	Yes	Proximal upper	No	No	N/A	No

*DVT*, Deep-vein thrombosis; *PE*, pulmonary embolism; *N/A*, not available.

## Supplementary Data

### Audio

Audio 1 This is an audio recording of the presentation of the associated abstract and discussion. The abstract presentation ends at 7:36 and the discussion begins at 7:48.

Video 1 Video available at: https://www.jtcvs.org/article/S2666-2736(26)00402-X/fulltext.
